# Brexpiprazole, a Serotonin-Dopamine Activity Modulator, Can Sensitize Glioma Stem Cells to Osimertinib, a Third-Generation EGFR-TKI, via Survivin Reduction

**DOI:** 10.3390/cancers11070947

**Published:** 2019-07-05

**Authors:** Shuhei Suzuki, Masahiro Yamamoto, Tomomi Sanomachi, Keita Togashi, Asuka Sugai, Shizuka Seino, Takashi Yoshioka, Chifumi Kitanaka, Masashi Okada

**Affiliations:** 1 Department of Molecular Cancer Science, Yamagata University School of Medicine, 2-2-2 Iida-nishi, Yamagata 990-9585, Japan; 2 Department of Clinical Oncology, Yamagata University School of Medicine, 2-2-2 Iida-nishi, Yamagata 990-9585, Japan; 3 Department of Ophthalmology and Visual Sciences, Yamagata University School of Medicine, 2-2-2 Iida-nishi, Yamagata 990-9585, Japan; 4 Research Institute for Promotion of Medical Sciences, Yamagata University Faculty of Medicine, 2-2-2 Iida-nishi, Yamagata 990-9585, Japan

**Keywords:** glioma stem cell, glioblastoma, brexpiprazole, osimertinib, survivin, xenograft

## Abstract

Glioblastoma is a primary brain tumor associated with a poor prognosis due to its high chemoresistance capacity. Cancer stem cells (CSCs) are one of the mechanisms of chemoresistance. Although therapy targeting CSCs is promising, strategies targeting CSCs remain unsuccessful. Abnormal activation of epidermal growth factor receptors (EGFRs) due to amplification, mutation, or both of the *EGFR* gene is common in glioblastomas. However, glioblastomas are resistant to EGFR tyrosine kinase inhibitors (EGFR-TKIs), and overcoming resistance is essential. Brexpiprazole is a new, safe serotonin-dopamine activity modulator used for schizophrenia and depression that was recently reported to have anti-CSC activity and function as a chemosensitizer. Here, we examined its chemosensitization effects on osimertinib, a third-generation EGFR-TKI with an excellent safety profile, in glioma stem cells (GSCs), which are CSCs of glioblastoma. Brexpiprazole treatment sensitized GSCs to osimertinib and reduced the expression of survivin, an antiapoptotic factor, and the pharmacological and genetic inhibition of survivin mimicked the effects of brexpiprazole. Moreover, co-treatment of brexpiprazole and osimertinib suppressed tumor growth more efficiently than either drug alone without notable toxicity in vivo. This suggests that the combination of brexpiprazole and osimertinib is a potential therapeutic strategy for glioblastoma by chemosensitizing GSCs through the downregulation of survivin expression.

## 1. Introduction

Glioblastoma is the most common primary brain tumor, accounting for 60% to 70% of glial brain tumors [1,2]. The outcome of glioblastoma is poor due to its highly infiltrative growth and high chemoresistance to therapeutic agents such as temozolomide [3,4]. Cancer stem cells (CSCs), which have high tumor initiation capacity and are resistant to chemotherapeutic reagents, play a role in chemoresistance [5,6,7,8]. Thus, the development of drugs eradicating glioma stem cells (GSCs), CSCs of glioblastoma, is important for the treatment of glioblastoma. However, the number of drugs used for glioblastoma is limited because drugs that poorly penetrate the blood–brain barrier (BBB) are not effective for glioblastoma [9].

The *EGFR* gene is amplified in 40% of glioblastomas [10,11]. Approximately 50% of *EGFR*-amplified glioblastomas express epidermal growth factor receptor (EGFR) variant III (EGFRvIII) resulting from the in-frame deletion of exons 2–7. EGFRvIII lacks a major part of the extracellular domain and is constitutively active [12,13]; therefore, EGFRvIII plays a role in the progression of glioblastoma [10,11,14]. Thus, inhibitors of EGFR are considered as a therapeutic option for glioblastoma [15,16,17]. However, in clinical trials with epidermal growth factor receptor tyrosine kinase inhibitors (EGFR-TKIs), such as gefitinib, erlotinib, and dacomitinib, clinical benefits were limited or absent [18,19,20,21]. Furthermore, the reason for resistance to EGFR-TKIs in glioblastoma remains unknown [15,19].

Osimertinib is an oral, third generation, irreversible EGFR-TKI. Osimertinib was developed to treat the “gatekeeper” *EGFR* mutation, which replaces a threonine at codon 790 with a methionine (T790M) that is acquired in approximately 50% of cases of non-small cell lung cancer (NSCLC) during treatment with first-generation TKIs [22,23]. Osimertinib is used as second-line chemotherapy for patients with metastatic NSCLC harboring T790M *EGFR* mutation who have disease progression during or after therapy with an EGFR-TKI. In a clinical trial, the efficacy and safety of osimertinib were superior to those of standard EGFR-TKIs in the first-line treatment of NSCLC, suggesting that osimertinib can be used for first-line chemotherapy [24]. Moreover, osimertinib had a better toxicity profile than standard EGFR-TKIs in clinical studies [23,24]. It can also penetrate the blood–brain barrier more efficiently than other EGFR-TKIs [25], and clinical trials demonstrated osimertinib to be more effective for NSCLC with brain metastasis than standard therapy [23,24,26]. Therefore, osimertinib is a potential candidate drug for the treatment of glioblastoma. However, the effects of osimertinib on glioblastoma have not been examined, and there are concerns that glioblastoma might be resistant to osimertinib, similar to other EGFR-TKIs.

Brexpiprazole is a new antipsychotic drug for depression and schizophrenia. Brexpiprazole was developed as a drug chemically and pharmacologically related to aripiprazole, a serotonin–dopamine activity modulator. Although brexpiprazole has similar pharmacological activity to aripiprazole, brexpiprazole has a better safety profile due to its lower intrinsic activity at the dopaminergic D2 and D3 receptors [27,28,29]. We previously reported that aripiprazole has anticancer effects and acts as a chemosensitizer in CSCs of NSCLC and pancreatic cancer [30]. Moreover, we recently revealed that brexpiprazole exhibits anticancer activity against several types of cancer, including glioblastoma, and chemosensitizes CSCs of pancreatic cancer and NSCLC to gemcitabine and 5-fluorouracil by downregulating survivin, an antiapoptotic protein [31]. Suppression of survivin is involved in the resistance to first-generation EGFR-TKIs of NSCLC: erlotinib and gefitinib [32,33,34]. These observations suggest that brexpiprazole acts as a chemosensitizer of EGFR-TKIs. Furthermore, as there are no clinically approved drugs targeting survivin, from the standpoint of clinical application, it is of interest to explore whether brexpiprazole, a clinically available and safe drug, chemosensitizes GSCs to osimertinib. Thus, in this study, we examined the combinational effects of osimertinib and brexpiprazole on GSCs in vitro and in vivo and addressed the mechanism of the combinational effects.

## 2. Results

### 2.1. Brexpiprazole Sensitizes GSCs to Osimertinib

**Figure 1:**
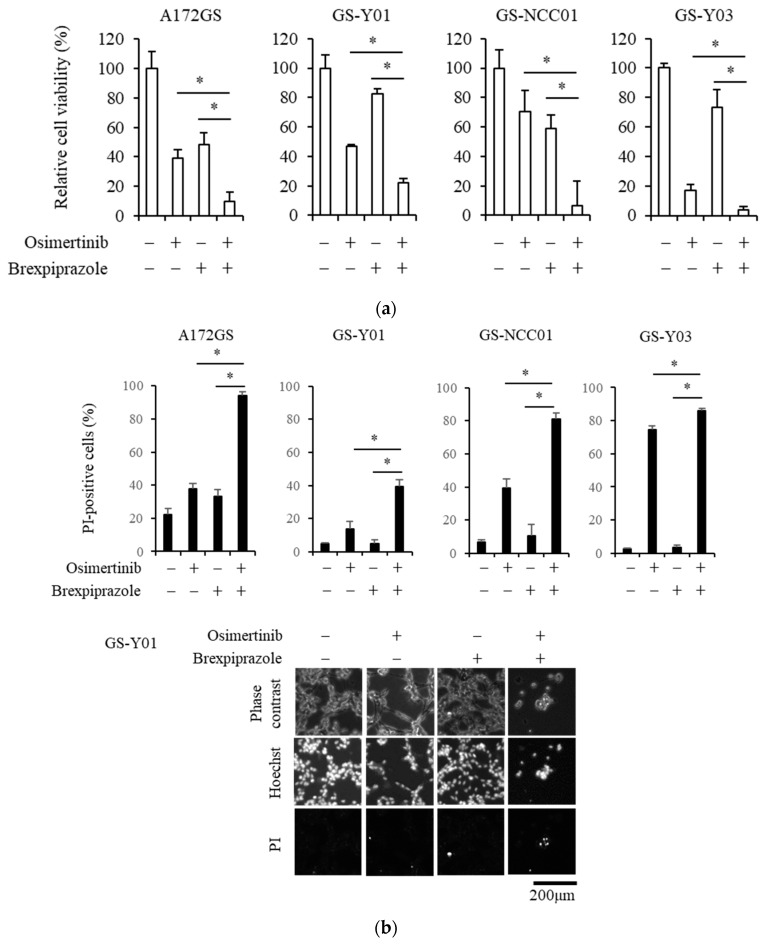

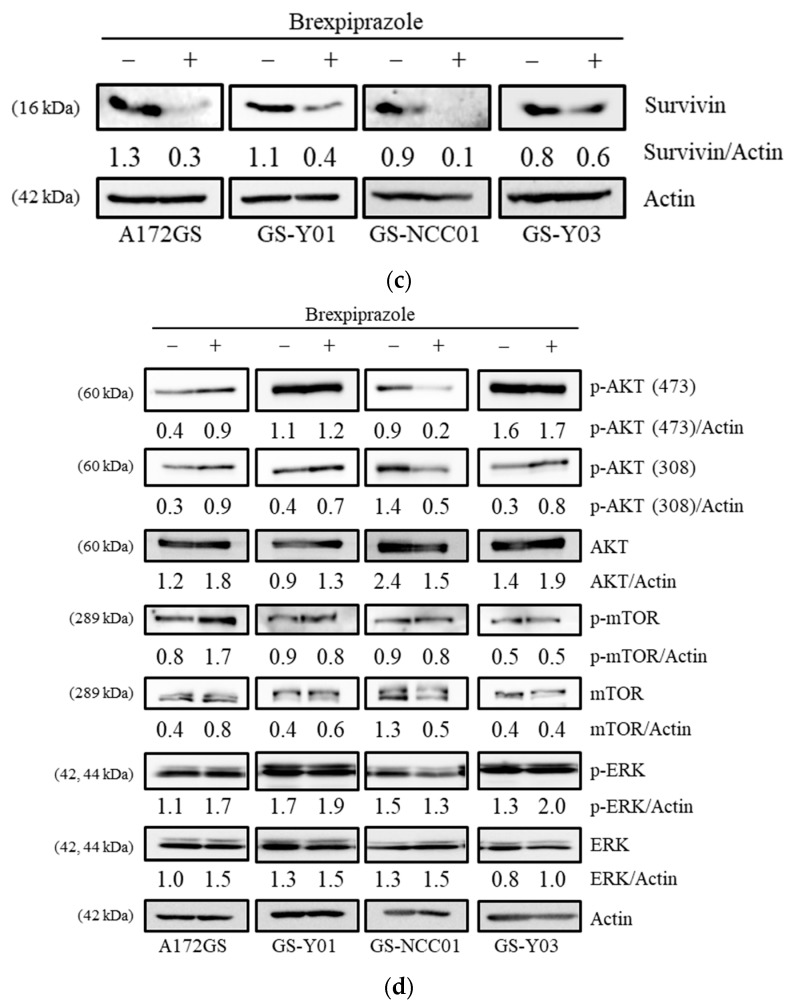
The effects of osimertinib and brexpiprazole combination treatment on glioma stem cell (GSC) proliferation and survivin expression: GSCs (A172GS, GS-Y01, GS-NCC01, and GS-Y03) were treated with or without 1.5 μM of osimertinib and with or without 3 μM of brexpiprazole for 3 days and were then subjected to several analyses. (**a**) The cells were subjected to cell viability assay using WST-8. Values represent means + SD from quadruplicate samples of a representative experiment repeated with similar results. * *p* < 0.05. (**b**) The cells were subjected to cell death assay using Hoechst 33342 (Hoechst) and propidium iodide (PI). The upper graphs show the proportion of dead cells as means + SD from triplicate samples of a representative experiment repeated with similar results. * *p* < 0.05. The lower panels show representative phase contrast images, Hoechst-positive images (total cells), and PI-positive cells (dead cells) among GS-Y01 cells. Scale bar: 200 μm. (**c,d**) The cells were subjected to immunoblot analysis for survivin, p-AKT, AKT, p-mTOR, mTOR, p-ERK, and ERK.

As previously reported, glioblastoma tumor-initiating cells have varying degrees of responsiveness to EGFR-TKIs [35]. We first examined the responsiveness of different types of GSCs to osimertinib using a representative GSC line, A172GS, and patient-derived GSC lines: GS-Y01, GS-NCC01, and GS-Y03 (Figure 1a,b). Regarding osimertinib treatment, GS-NCC01 exhibited low responses, A172GS and GS-Y01 exhibited moderate responses, and GS-Y03 exhibited high responses, indicating different sensitivities to osimertinib by these GSCs. As the presence of EGFRvIII mutations may affect the sensitivity of osimertinib, we examined the expression of EGFRvIII in these cells. A172GS and GS-NCC01 cells expressed EGFRvIII but GS-Y01 and GS-Y03 cells did not, suggesting no correlation between EGFRvIII expression and osimertinib sensitivity (Figure S1). Next, we examined whether brexpiprazole increases the responsiveness to osimertinib of the GSCs. The combination of brexpiprazole with osimertinib reduced the viable cell number (Figure 1a) and increased cell death (Figure 1b) in the GSCs irrespective of their sensitivity to osimertinib. As we previously reported that brexpiprazole reduces the expression of survivin, a chemoresistant factor of cancer cells [30], in CSCs from different cancers, including GS-Y03 cells [31], we assessed the alteration of survivin expression by brexpiprazole in these cells. Brexpiprazole reduced the expression of survivin in the GSCs (Figure 1c). Furthermore, we examined whether brexpiprazole affects downstream signals of EGFR. However, brexpiprazole treatment did not cause consistent changes in the expression levels of p-AKT, p-mTOR, and p-ERK among the cell lines examined (Figure 1d).

### 2.2. Pharmacological Inhibition of Survivin Sensitizes GSCs to Osimertinib

**Figure 2:**
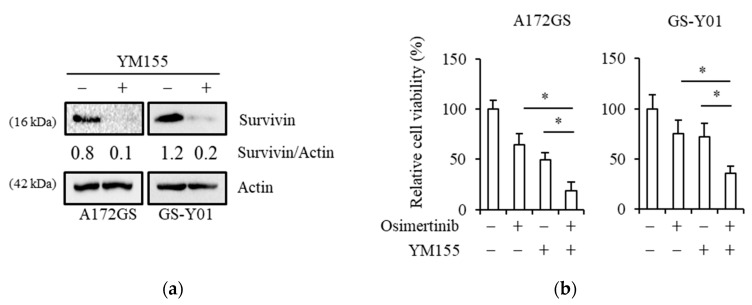
Pharmacological inhibition of survivin by YM155-induced sensitization of GSCs to osimertinib: The indicated GSCs were treated with or without 10 nM of YM155 and with or without 1.5 μM of osimertinib for 3 days. (**a**) The cells (with or without YM155 only) were subjected to immunoblot analysis for survivin protein expression. (**b**) The cells were then subjected to cell viability assay using WST-8. Values represent means +SD from quadruplicate samples of a representative experiment repeated with similar results. * *p* < 0.05.

As recent studies revealed that EGFR-TKI resistance is partially due to the expression of survivin, a representative antiapoptotic molecule [32,33,34], we evaluated the effects of the pharmacological inhibition of survivin on two representative GSC lines, A172GS and GS-Y01, which are moderately resistant to osimertinib using YM155, a pharmacological inhibitor of survivin [36,37]. YM155 treatment reduced the expression of survivin (Figure 2a). Next, we examined whether YM155 treatment mimics the effects of brexpiprazole as a sensitizer to osimertinib. YM155 treatment sensitized the GSCs to osimertinib to a similar degree as brexpiprazole (Figure 2b), suggesting the involvement of survivin in the mechanism of osimertinib sensitization by brexpiprazole.

### 2.3. Genetic Inhibition of Survivin Mimics Brexpiprazole Treatment

**Figure 3:**
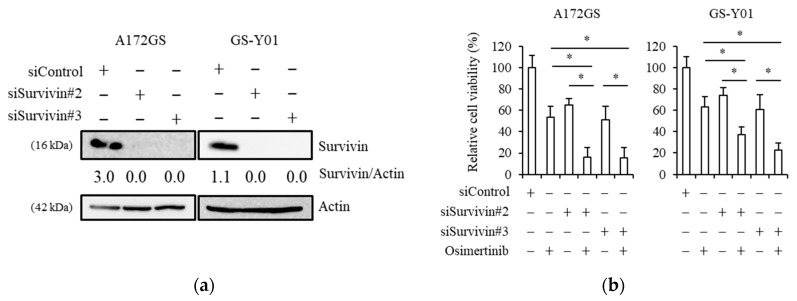
siRNA-mediated knockdown of survivin sensitizes GSCs to osimertinib: The indicated GSCs were transfected with a non-targeting siRNA (siControl) or either of the siRNAs against survivin (siSurvivin#2 or siSurvivin#3) for 3 days. (**a**) The cells were subjected to immunoblot analysis for survivin protein expression. (**b**) Then, the transfected cells were treated with or without 1.5 μM of osimertinib for 3 days, and the cells were subjected to cell viability assay using WST-8. Values represent means + SD from quadruplicate samples of a representative experiment repeated with similar results. * *p* < 0.05.

To exclude the possibility of off-target effects of brexpiprazole and YM155 on mechanisms other than survivin, we next examined the effects of genetic inhibition of survivin on the responsiveness to osimertinib by the GSCs using siRNA. After introducing two different siRNAs against survivin, siSurvivin#2 and siSurvivin#3, the expression of survivin decreased (Figure 3a). On the other hand, a non-targeting siRNA (siControl) did not cause a decrease in survivin expression. In the cells with siRNAs against survivin, osimertinib resistance was attenuated (Figure 3b). Therefore, survivin expression is necessary for osimertinib resistance in GSCs. 

### 2.4. Brexpiprazole Sensitizes GSCs to Osimertinib in Vivo.

**Figure 4:**
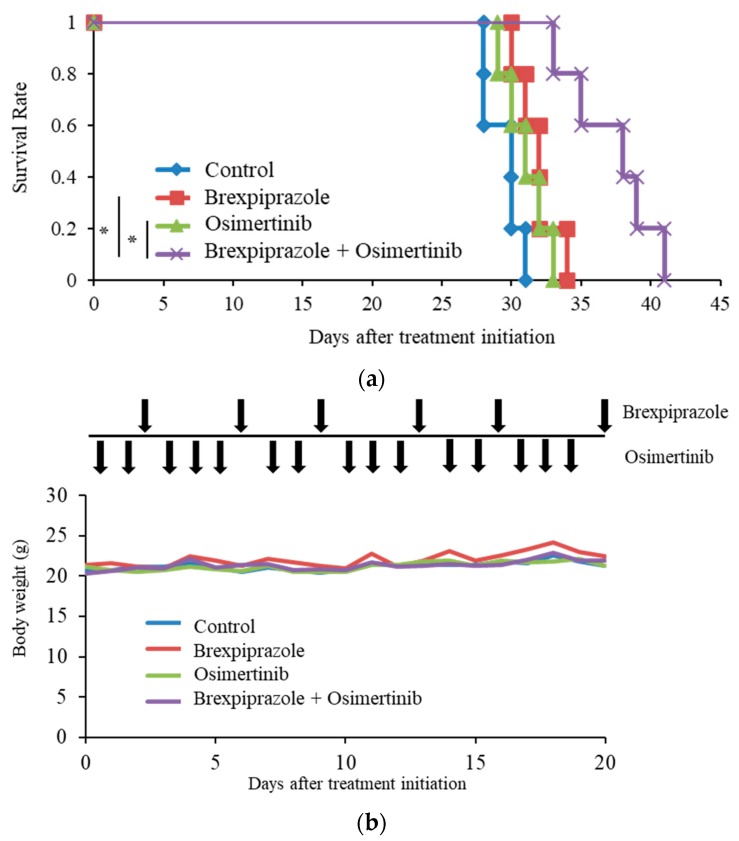
Systemic combination administration of brexpiprazole and osimertinib has anticancer stem effects in vivo. After patient-derived GSCs (GS-Y03, 1 × 10^4^ cells) were intracranially injected to the left-brain hemisphere of nude mice, the indicated drugs (3 mg/kg of brexpiprazole twice a week and 5 mg/kg of osimertinib 5 times a week) were orally administered after 2 days. (**a**) Survival was evaluated by a Kaplan–Meier analysis. (**b**) The body weights of the mice (means with SD) were presented. Arrows indicate the treatment schedule. * *p* < 0.05.

Survivin inhibition effectively attenuated the resistance to osimertinib of the GSCs in vitro. To adapt the combination of brexpiprazole and osimertinib to clinical settings, we examined the efficacy of the combination in a preclinical mouse xenograft model. GS-Y03 cells were implanted intracranially into nude mice, and the mice were treated with osimertinib, brexpiprazole, their combination, or solvent control. As a result, the mice treated with the combination of osimertinib and brexpiprazole survived significantly longer than those treated with either osimertinib or brexpiprazole (Figure 4a). Administration of brexpiprazole and osimertinib did not reduce the body weight of the mice (Figure 4b). No notable adverse effects were observed in the mice.

## 3. Discussion

Glioblastoma is one of the most intractable malignancies. Although surgery followed by radiation or chemotherapy improves the prognosis, the effectiveness of these therapies is limited, resulting in a median survival of only 12–15 months [3,38,39,40]. One of the reasons for the ineffectiveness of the therapies is GSCs. GSCs have a high tumor initiation capacity and are resistant to chemotherapy and radiotherapy [41]. Therefore, it is essential to eradicate GSCs to cure glioblastomas. As the *EGFR* gene is amplified in 40% of primary glioblastomas [10,11], therapy targeting EGFR was considered promising. However, the results of clinical trials with first- and second-generation EGFR-TKIs for glioblastoma were disappointing [18,19,20]. Osimertinib, an oral third-generation EGFR-TKI, penetrates the BBB more effectively [25] and has a better safety profile than other EGFR-TKIs [23,24]. A clinical trial with osimertinib on glioblastoma is still ongoing (NCATS 1-UH2-TR001370-01), and the effectiveness of osimertinib remains unknown, even for in vitro studies. In this study, we found that the sensitivity to osimertinib of GSCs was different among the four types of GSCs examined. Of the four, three types of GSCs were resistant and only one type was sensitive to osimertinib, demonstrating that GSCs are often resistant to osimertinib. Brexpiprazole was developed as a new serotonin-dopamine activity modulator with an improved safety profile to succeed aripiprazole. We recently reported that brexpiprazole acts as a chemosensitizer to gemcitabine and 5-fluorouracil in CSCs of pancreatic cancer and NSCLC [31]. In this study, we revealed that brexpiprazole sensitized the GSCs to osimertinib regardless of their sensitivity to osimertinib in vitro. Moreover, brexpiprazole sensitized the osimertinib-resistant GSCs to osimertinib in a preclinical mouse model.

The mechanisms of resistance to EGFR-TKI in glioblastomas remain obscure [18,19,20,21]. The pharmacological efficacy of gefitinib and erlotinib, first-generation EGFR-TKIs, mainly depends on activation mutation in exons 19 and 21 of the tyrosine kinase domain. The absence of these mutations in glioblastoma partially explains the resistance to gefitinib and erlotinib [15,42]. Another possible mechanism is an alternative activating signal that compensates for the inactivation of EGFR signaling by EGFR-TKIs [43]. It was previously reported that the absence of EGFRvIII and loss of PTEN are partial determinants of resistance [15,44]. Inhibition of mTOR, a downstream molecule of the PI3 kinase/PTEN/AKT pathway, promoted the response of glioma cells to EGFR-TKIs in vitro [45,46]. However, phase II clinical trials with relapsed glioblastoma patients did not support the correlation between the responsiveness to erlotinib and the expression of EGFRvIII and PTEN [47,48]. Moreover, the combination of sirolimus, an inhibitor of mTOR, and EGFR-TKIs did not improve the responsiveness in patients with recurrent glioblastomas [49]. Alternatively, inhibition of EGFR by erlotinib in EGFRvIII-expressing U87 glioblastoma cells may increase the expression of PDGFRβ, which compensates for signaling inhibited by erlotinib. Co-inhibition of PDGFRβ by AG1295, an inhibitor of PDGFR, with erlotinib effectively suppressed tumor growth [50]. Erlotinib upregulates promyelocytic leukemia (PML) protein, which is a negative regulator of AKT-mTOR signaling, to promote resistance to erlotinib, and treatment with a PML inhibitor chemosensitizes glioblastoma cells to erlotinib and an mTOR inhibitor, suggesting the clinical relevance of the combination of PML-targeting drugs and erlotinib [51]. Screening with an shRNA library using glioblastoma cells revealed that the combined inhibition of dopamine D2 receptors and EGFR-TKI leads to synergistic tumoricidal activity through suppression of the MAP kinase pathway [52]. Although the mechanism they suggested is different from ours, those data support our findings that brexpiprazole sensitized GSCs to osimertinib.

We recently reported that brexpiprazole reduces the expression of survivin and chemosensitizes pancreatic cancer and NSCLC to gemcitabine and 5-fluorouracil [31]. However, whether brexpiprazole chemosensitizes GSCs to EGFR-TKIs via the downregulation of survivin was unclear. In this study, both the genetic inhibition of survivin by siRNA and the pharmacological inhibition by YM155, a suppressor of survivin, chemosensitized GSCs to osimertinib. Although our data do not exclude the possibility of involvement of mechanisms other than survivin, they do suggest that downregulation of survivin is, at least in part, one of the major mechanisms of chemosensitization by brexpiprazole. Consistent with our data, survivin is involved in resistance against EGFR-TKIs in NSCLCs [32,33,34]. Clinical benefits of YM155 are not proven in clinical trials [53,54,55], and YM155 is not approved worldwide. As brexpiprazole is FDA-approved and applicable for clinical translation with a detailed safety profile [28,56,57], it is a good candidate drug to chemosensitize GSCs to EGFR-TKIs by downregulating the expression of survivin.

In a clinical trial, serious adverse events of grade 3 or higher were reported in fewer patients administered osimertinib than in those receiving standard EGFR-TKIs (34% vs. 45%), suggesting the better safety profile of osimertinib [24]. Brexpiprazole was developed to succeed aripiprazole, a serotonin–dopamine activity modulator. Compared with aripiprazole, brexpiprazole was reported to cause fewer adverse effects; akathisia and insomnia occur 50% less frequently than with aripiprazole [28,58,59]. Of note, the combination of brexpiprazole and osimertinib exhibited therapeutic effects in mice but did not cause any notable adverse effects, including alteration of body weight, demonstrating the clinical relevance of our animal experiments.

## 4. Materials and Methods

### 4.1. Antibodies and Reagents

Anti-survivin (#2808), anti-p-AKT (Ser473 #9271, Thr308 #9275), anti-AKT (#9272), anti-p-mTOR (#2974), anti-mTOR (#2972), anti-p-ERK (#9101), anti-ERK (#4695), and anti-EGFR (#4267) antibodies were purchased from Cell Signaling Technology, Inc. (Beverly, MA, USA). Anti-β-actin (A1978) antibody was from Sigma (St. Louis, MO, USA). Osimertinib and YM155 were purchased from Chemscene LLC. (Monmouth Junction, NJ, USA), and dissolved in dimethyl sulfoxide (DMSO) to 10 mM and 20 μM, respectively, as stock solutions. Brexpiprazole was from Cayman Chemical Company (Ann Arbor, MI, USA) and was dissolved in DMSO to 10 mM as a stock solution.

### 4.2. Cell Culture

Patient-derived GSCs used in this study (GS-Y01, GS-NCC01, and GS-Y03) were established and cultured as previously described [60,61], and the A172GS GSC line was also established and cultured as previously described [62]. In brief, cells were cultured on collagen-I-coated dishes (IWAKI, Tokyo, Japan) in stem cell culture medium [60] (DMEM/F12 medium with 1% B27 (Thermo Fisher Scientific, Waltham, MA, USA), 20 ng/mL of EGF and FGF2 (Peprotech, Inc., Rocky Hill, NJ, USA), D-(+)-glucose (final concentration of 26.2 Mm), L-glutamine (final concentration of 4.5 mM), 100 units/mL of penicillin, and 100 µg/mL of streptomycin). This stem cell culture medium was replaced every 3 days, and EGF and FGF2 were added to the medium every day. 293T cells were cultured in DMEM supplemented with 10% fetal bovine serum, 100 units/mL of penicillin, and 100 µg/mL of streptomycin.

### 4.3. Cell Viability and Cell Death Assays

Cell viability assays were performed as previously described [63]. In brief, cell viability was assessed by the tetrazolium salt reduction method using WST-8 (Cell Counting Kit-8; Dojindo Laboratories, Kumamoto, Japan) according to the manufacturer’s protocol. Cells (500–1000 cells/well) plated in 96-well collagen I-coated plates were treated with drugs the next day for 3 days. WST-8 reagent was then added, and the cells were incubated for 1–3 h at 37 °C. Absorbance at 450 nm was measured using a microplate reader (Model 680, Bio-Rad, Hercules, CA, USA). Relative cell viability was calculated as a percentage of the absorbance of treated samples relative to that of control samples. Cell viability assays were performed in four replicates. Alternatively, cells were incubated in situ with propidium iodide (PI, 1 μg/mL) and Hoechst 33,342 (10 μg/mL) for 5 min at 37 °C in the CO_2_ incubator to stain dead cells and the cell nuclei, respectively. Then, the numbers of PI- and Hoechst-stained cells were counted using a fluorescence microscope (CKX41; Olympus, Tokyo, Japan), and the percentage of PI-stained cells (dead cells) against Hoechst-stained cells (total cells) was calculated [64].

### 4.4. Immunoblot Analysis

Cells were washed with ice-cold PBS and lysed in RIPA buffer (10 mM Tris-HCl (pH 7.4), 0.1% SDS, 0.1% sodium deoxycholate, 1% NP-40, 150 mM NaCl, 1 mM EDTA, 1.5 mM Na_3_VO_4_, 10 mM NaF, 10 mM sodium pyrophosphate, 10 mM sodium β-glycerophosphate. and 1% protease inhibitor cocktail set III (Wako Pure Chemical Industries, Ltd, Osaka, Japan)). After centrifugation for 10 minutes at 14,000× *g* at 4 °C, the supernatants were harvested as the cell lysates and the protein concentration of the cell lysates was measured using the BCA protein assay kit (Thermo Fisher Scientific). Cell lysates containing equal amounts of protein were separated by SDS-PAGE and transferred to polyvinylidene difluoride membranes. The membranes were probed with primary antibodies and then with an appropriate HRP-conjugated secondary antibody according to the manufacturer’s protocol. Immunoreactive bands were visualized by Immobilon Western Chemiluminescent HRP Substrate (Merck Millipore, Billerica, MA, USA) and detected semi-quantitatively using a ChemiDoc Touch Imaging System (Bio-Rad). The relative density of immunoreactive bands was analyzed by densitometry using ImageJ 1.52a software (National Institutes of Health, Bethesda, MD, USA). The original immunoblot pictures were shown in Figure S2.

### 4.5. Gene Silencing with siRNA.

Two siRNAs against human survivin (*BIRC5,* #2; HSS 179404 and #3; HSS 179405) and Medium GC Duplex #2 of Stealth RNAi^TM^ siRNA Negative Control Duplexes (non-targeting control) were purchased from Thermo Fisher Scientific. Cells were transiently transfected with RNAs using Lipofectamine RNAiMAX^TM^ (Thermo Fisher Scientific) according to the manufacturer’s directions.

### 4.6. Mouse Study

After the nude mice (7-week-old male BALB/cAJcl-nu/nu mice (CLEA Japan, Inc., Tokyo, Japan)) were anesthetized by subcutaneous injection of 25 mg/kg of midazolam, 5 mg/kg of medetomidine, and 25 mg/kg of butorphanol, GS-Y03 (1 × 10^4^ cells diluted in 10 μL of DMEM/F12 medium), patient-derived GSCs, were stereotactically injected into the left cerebral hemisphere at a depth of 3 mm, as previously described [62]. Two days after intracranial implantation, brexpiprazole was administered by oral gavage twice a week (dose of 3 mg/kg, final volume 100 μL in DMSO) and osimertinib was administered by oral gavage 5 times a week (dose of 5 mg/kg, final volume 100 μL in DMSO). Control mice were administered the same volume of DMSO. Five mice were assigned to each group. All mice were monitored for their general health status. The termination criteria were marked weight loss of greater than 20% of that at the initiation of the experiment or apparent health problems such as inability to access food and water. The experiment was approved by the Animal Research Committee of Yamagata University (30027, 14 March 2018).

### 4.7. Transfection of Plasmid

The plasmid pT3.5-CMV-EGFRvIII was a gift from John Ohlfest (Addgene plasmid #20280). The plasmid was transfected into 293T cells by using Lipofectamine 2000 (Thermo Fischer Scientific) according to the manufacturer’s instructions. The cell lysate from the transfected cells was used as a positive control for EGFRvIII.

### 4.8. Statistical Analysis

Results are expressed as the means and standard deviation (SD). The differences were compared by the two-tailed *t*-test. The survival curve was evaluated by the Kaplan–Meier method and analyzed using the log-rank test. *p*-values < 0.05 were considered significant and indicated with asterisks. 

## 5. Conclusions

In conclusion, brexpiprazole, a safe and newly-developed antipsychotic drug, sensitized GSCs to osimertinib in vitro and in vivo via survivin downregulation.

## Supplementary Materials

The following is available online at https://www.mdpi.com/2072-6694/11/7/947/s1: Figure S1: Expression of EGFR in GSCs: The expression of EGFR in the GSCs was examined by immunoblot analysis. The expression of EGFRvIII was evaluated by comparing with sample of 293T cells transfected with a plasmid for EGFRvIII overexpression. Figure S2: Original immunoblotting membranes.
