# Supplementary material for: Brexpiprazole, a Serotonin-Dopamine Activity Modulator, Can Sensitize Glioma Stem Cells to Osimertinib, a Third-Generation EGFR-TKI, via Survivin Reduction
Source: Cancers (Basel). 2019 Jul 5;11(7):947. doi: 10.3390/cancers11070947 (PMC6679129; doi:10.3390/cancers11070947)
Supplement: Supplementary file 1 [file cancers-11-00947-s001.pdf]

Supplementary Materials: Brexpiprazole, A Serotonin-Dopamine Activity Modulator, Can Sensitize Glioma Stem Cells to Osimertinib, A Third-Generation EGFR-TKI, via Survivin Reduction

Shuhei Suzuki, Masahiro Yamamoto, Tomomi Sanomachi, Keita Togashi, Asuka Sugai, Shizuka Seino, Takashi Yoshioka, Chifumi Kitanaka and Masashi Okada

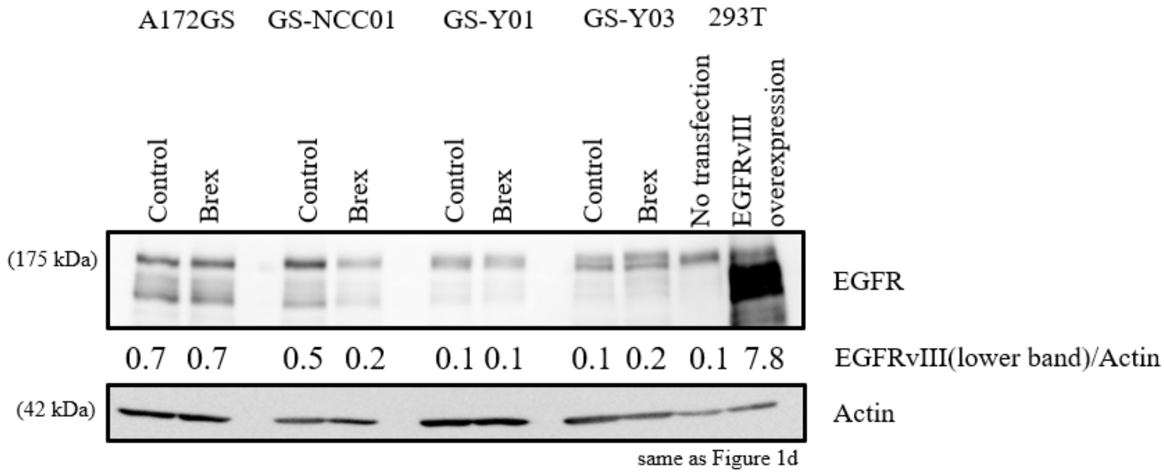

Figure S1. Expression of epidermal growth factor receptor (EGFR) in glioma stem cells (GSCs). The expression of EGFR in the GSCs was examined by immunoblot analysis. The expression of EGFR variant III (EGFRvIII) was evaluated by comparing with sample of 293T cells transfected with a plasmid for EGFRvIII overexpression.

(Figure 1c)

Survivin (A172GS, GS-NCC01) Survivin (GS-Y01) Survivin (GS-Y03)

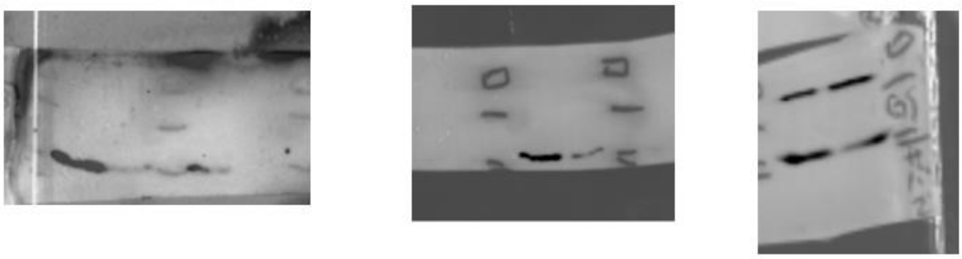

Actin (A172GS, GS-NCC01) Actin (GS-Y01) Actin (GS-Y03)

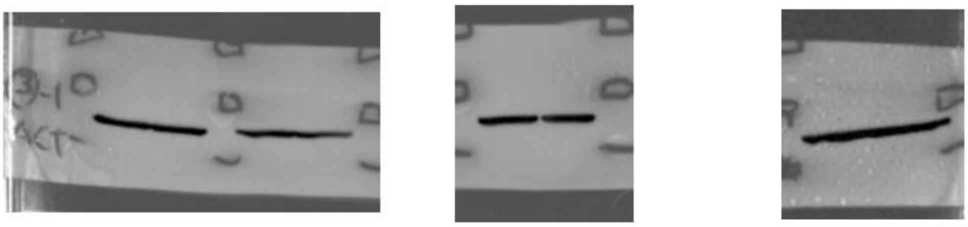

(Figure 1d)

p-AKT (473) (A172GS) p-AKT (473) (GS-Y01) p-AKT (473) (GS-NCC01) p-AKT (473) (GS-Y03)

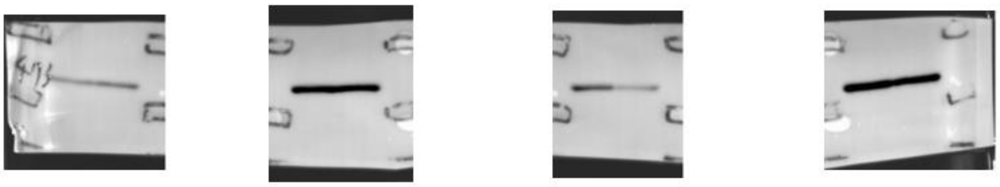

p-AKT (308) (A172GS) p-AKT (308) (GS-Y01) p-AKT (308) (GS-NCC01) p-AKT (308) (GS-Y03)

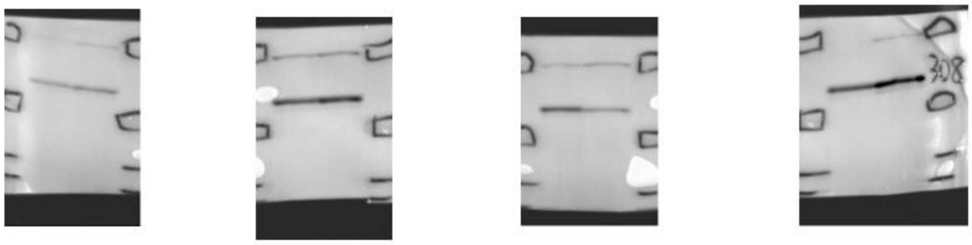

AKT (A172GS) AKT (GS-Y01) AKT (GS-NCC01) AKT (GS-Y03)

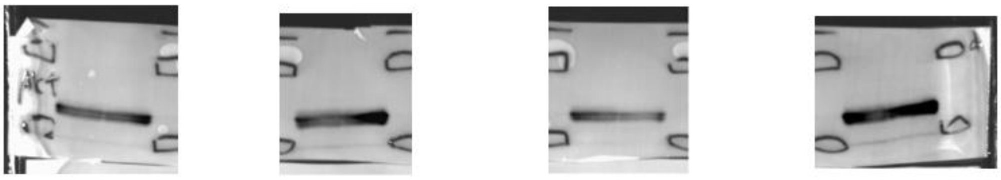

p-mTOR (A172GS) p-mTOR (GS-Y01) p-mTOR (GS-NCC01) p-mTOR (GS-Y03)

p-mTOR (A172GS)

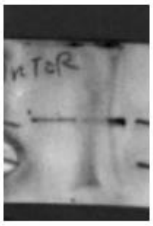

p-mTOR (GS-Y01)

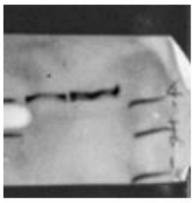

p-mTOR (GS-NCC01)

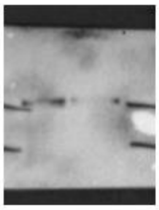

p-mTOR (GS-Y03)

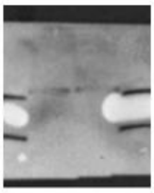

mTOR (A172GS)

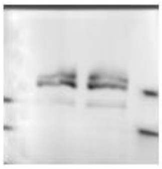

mTOR (GS-Y01)

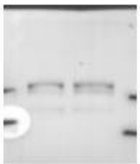

mTOR (GS-NCC01)

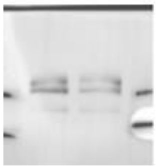

mTOR (GS-Y03)

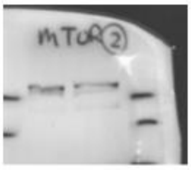

(Figure 1d)

p-ERK (left to right, A172GS, GS-NCC01, GS-Y01, GS-Y03)

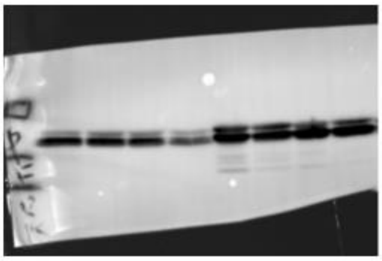

ERK (left to right, A172GS, GS-NCC01, GS-Y01, GS-Y03)

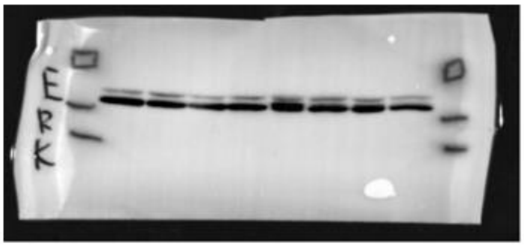

Actin (A172GS)

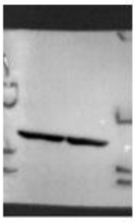

Actin (GS-Y01)

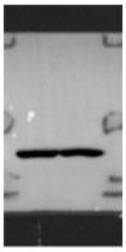

Actin (GS-NCC01)

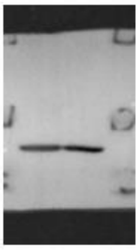

Actin (GS-Y03)

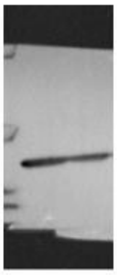

(Figure 2)

Survivin (A172GS) Actin (A172GS) Survivin (GS-Y01) Actin (GS-Y01)

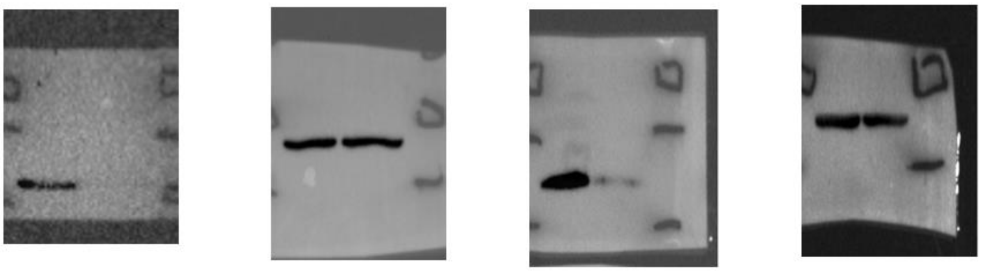

(Figure 3)

Survivin (A172GS) Actin (A172GS) Survivin (GS-Y01) Actin (GS-Y01)

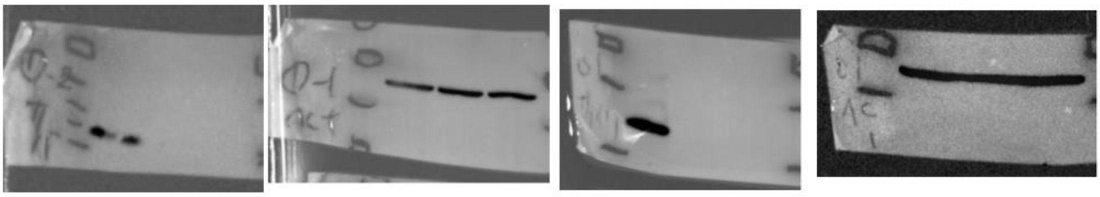

(Figure S1)

EGFR

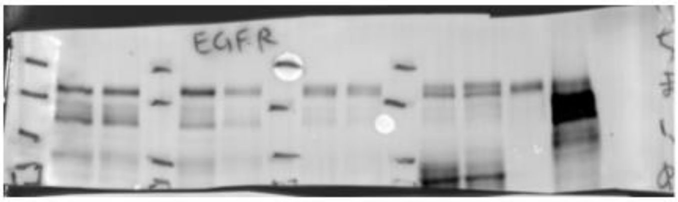

Actin

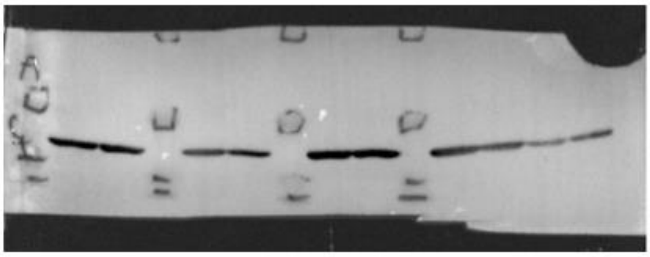

same as Figure 1d

Figure S2. Immunoblotting membranes.

© 2019 by the authors. Licensee MDPI, Basel, Switzerland. This article is an open access article distributed under the terms and conditions of the Creative Commons Attribution (CC BY) license (http://creativecommons.org/licenses/by/4.0/).
